# Dressler syndrome and post-cardiac injury syndrome: an autoinflammatory framework for reappraising a seventy-year-old diagnosis: a state-of-the-art review

**DOI:** 10.3389/fimmu.2026.1977374

**Published:** 2026-10-20

**Authors:** Yashendra Sethi, Arsalan Moinuddin, Kunal Mahajan, Amit Sharma, Chris Pavlovic, Thomas Pavlovic, Giuseppe Biondi-Zoccai

**Affiliations:** 1 Department of Cardiometabolic Sciences, Nexoria Institute of Cardiometabolic Excellence (NICE), Vigyaved Healthcare, Dehradun, India; 2 Department of Cardiology, PearResearch, Dehradun, India; 3 Department of Medicine, Subharti Medical College, Swami Vivekanand Subharti University, Meerut, India; 4 Department of Physiology, Shri Guru Ram Rai Institute of Medical Sciences, Dehradun, India; 5 Department of Cardiology, Himachal Heart Institute, Mandi, India; 6 Department of Cardiology, Subharti Medical College, Swami Vivekanand Subharti University, Meerut, India; 7 Department of Cardiology, Northshore Heart, Lutheran General Hospital, Park Ridge, IL, United States; 8 Department of Medical-Surgical Sciences and Biotechnologies, Sapienza University of Rome, Latina, Italy; 9 IRCCS Maria Cecilia Hospital – GVM Care & Research, Cotignola, Italy

**Keywords:** Dressler syndrome, Interleukin-1, NLRP3 inflammasome, Pericarditis, Post-cardiac injury syndrome

## Abstract

Dressler syndrome, the delayed pericarditis after myocardial infarction remains firmly established in cardiological teaching, yet contemporary direct evidence specific to it is sparse. Its reported incidence has fallen markedly in the reperfusion era, while post-cardiac injury syndrome (PCIS) is now encountered chiefly after cardiac surgery and catheter-based procedures. These trends reflect a change in the clinical settings in which delayed post-injury pericardial inflammation is recognised, rather than evidence of a transfer of the same biological disease burden; incidence estimates across eras and procedures are not directly comparable. The historical anti-heart antibody framework does not, on its own, account for current observations. Randomised trials in predominantly idiopathic recurrent pericarditis show that colchicine and interleukin-1 (IL-1)–directed agents (anakinra, rilonacept, goflikicept) reduce recurrence, and experimental and human pericardial tissue data implicate the NLRP3 inflammasome. These data support an important role for innate immune signalling in pericarditis, but they do not establish IL-1 dependency in late post-infarction Dressler syndrome, and they do not exclude a contribution of adaptive immunity to amplification, persistence or recurrence. We grade the evidence at three levels—direct human PCIS data, data from other human pericarditis phenotypes, and experimental data—and stratify therapeutic evidence by phenotype and drug class, distinguishing colchicine, for which randomised prevention data exist in post-pericardiotomy syndrome, from IL-1 blockade, for which no PCIS-specific trial exists. We propose a pericardial injury-threshold model as a testable hypothesis in which clinical expression reflects the interaction of damage-associated molecular pattern load, the directness of pericardial and mesothelial exposure, and host inflammatory susceptibility, with adaptive responses acting as potential amplifiers. The model yields falsifiable predictions and identifies a key unanswered question: whether IL-1 inhibition during infarct healing is safe and effective. We outline a staged research programme, including a candidate two-tier research case definition requiring prospective validation, registry-based ascertainment, pericardial fluid immunophenotyping, inflammasome genetics, and phenotype-specific trials, including of emerging direct NLRP3 inhibitors.

## 1 A diagnosis that outlived its evidence

In April 1956 William Dressler, working at Maimonides Hospital in Brooklyn, published ten patients in whom myocardial infarction was followed, after an interval of days to weeks, by fever, pleuritic pain, a friction rub, leucocytosis and pericardial or pleural effusion. He was careful about what he claimed. He called it a preliminary report, and he described the illness by analogy — as a complication resembling idiopathic, recurrent, benign pericarditis (1). Three years later he returned with forty-four cases and a fuller account, including the observation, easy to overlook and difficult to explain on any purely mechanical theory, that the syndrome relapsed (2). Seven decades later, Dressler syndrome—here used specifically to denote delayed post-myocardial-infarction pericarditis—has become an uncommon but still recognised manifestation within the broader spectrum of post-cardiac injury syndrome (PCIS).

The reason for its retreat is not mysterious, and it is a credit to the field. Timely reperfusion limits the mass of necrotic myocardium available to inflame the overlying pericardium, and the reported incidence of late post-infarction pericarditis has fallen substantially in the reperfusion era (3–5). By 2004 an editorial could ask, without irony, whether Dressler syndrome was dead (5). Subsequent case reports have nevertheless confirmed that delayed post-infarction pericarditis still occurs (6).

The decline of Dressler syndrome, however, does not imply that post-cardiac injury inflammation has disappeared from contemporary cardiovascular practice. PCIS is an umbrella construct encompassing inflammatory pericardial syndromes that follow myocardial infarction, cardiac surgery, catheter ablation, device implantation, and other forms of cardiac injury. Across these settings, post-procedural PCIS or pericarditis is reported more frequently than contemporary late post-infarction pericarditis, although direct quantitative comparisons are limited by major differences in case definitions, surveillance intensity, procedural populations, follow-up, and denominators (7–10). Accordingly, the shift is best understood as a change in the clinical context in which post-injury pericardial inflammation is now recognized, rather than evidence that its total biological burden has necessarily increased. Meanwhile the mechanistic framework for pericardial inflammation has evolved substantially, although much of the relevant evidence derives from recurrent or idiopathic pericarditis rather than Dressler syndrome itself. Randomized withdrawal trials of anakinra and rilonacept in recurrent pericarditis demonstrated marked reductions in recurrence among selected patients with inflammatory, recurrence-prone disease; these findings establish a central therapeutic role for interleukin-1 signalling in recurrent pericarditis, but they should not be interpreted as direct mechanistic evidence for late post-infarction Dressler syndrome (11, 12). Colchicine, which modulates microtubule-dependent inflammatory pathways and can inhibit inflammasome activation, has reduced recurrence across five randomized trials spanning acute, recurrent and post-surgical disease (13–18). A murine model has reproduced features of pericardial inflammation and responsiveness to targeted anti-inflammatory therapy, and human pericardium taken during flares shows inflammasome components upregulated (19). Yet the eponymous condition itself sits in a comparatively sparse contemporary evidence base for something so familiar. We identified no prospective cohort designed specifically around contemporary Dressler syndrome, and the condition has not served as a dedicated enrolment phenotype in randomised therapeutic trials. It does not appear in the adjudicated adverse-event lexicon of most infarction or ablation registries, which means that even its present-day incidence is often estimated indirectly rather than measured using a standardised prospective definition. Its management is therefore largely extrapolated from broader PCIS populations and from acute and recurrent pericarditis studies. This review sets out what is genuinely known, distinguishes evidence derived directly from PCIS from that extrapolated from other pericarditis phenotypes, proposes a testable mechanistic framework rather than an established replacement model, and argues that the remaining questions are amenable to prospective study if case definitions, phenotyping, and follow-up are standardised.

### 1.1 Literature search and evidence grading

This is a narrative state-of-the-art review, not a systematic review. To make the basis of our statements transparent, we searched PubMed/MEDLINE, Embase, Scopus and Web of Science from inception to 30 September 2026, supplemented by ClinicalTrials.gov and by the reference lists of retrieved articles and of the 2015 ESC, 2025 ESC and 2025 ACC pericardial guidance documents. Search terms combined condition terms (“Dressler syndrome,” “post-myocardial infarction syndrome,” “post-cardiac injury syndrome,” “postpericardiotomy syndrome,” “post-ablation pericarditis,” “pericarditis”) with mechanistic, therapeutic and epidemiological terms (“NLRP3,” “inflammasome,” “interleukin-1,” “autoinflammat*,” “anti-heart antibod*,” “colchicine,” “anakinra,” “rilonacept,” “goflikicept,” “incidence,” “cardiac magnetic resonance”). We considered randomised trials, cohort and registry studies, human tissue and fluid studies, experimental studies, guidelines and reviews published in English. Statements that we “identified no study” of a given design refer to this search. They describe the searched literature and are not claims of definitive absence. Evidence is graded at three levels throughout: Level A, direct evidence in human PCIS (studies enrolling patients with Dressler syndrome, post-pericardiotomy syndrome or post-procedural pericarditis); Level B, human evidence from other pericarditis phenotypes, predominantly idiopathic or recurrent; and Level C, evidence from experimental models or indirect human data, such as studies of interleukin-1 biology in myocardial infarction without pericarditis.

## 2 How the syndrome was built, and where the nosology went wrong

Dressler was not working in isolation. Two years after his preliminary report, Ito and Engle described a strikingly similar febrile pleuropericardial illness following surgery for non-rheumatic heart disease (20), and by 1965 Drusin and colleagues had defined the post-pericardiotomy syndrome in a six-year epidemiological study (21). The clinical resemblance prompted investigation of a potentially shared immunological mechanism, although the available evidence was largely associative.

Engle and colleagues showed, in prospective and triple-blind designs, that high-titre heart-reactive antibodies, measured using indirect immunofluorescence against cardiac tissue substrates, appeared in essentially every patient who developed post-pericardiotomy syndrome and tracked the clinical course (Level A, associative) (22, 23). In a paediatric cohort of four hundred, the syndrome occurred in 27 per cent, with a steep age gradient — only 3.5 per cent among infants under two years — and high-titre antibody in all affected children (24). These historical “anti-heart” or “anti-myocardial” antibody measurements should not be interpreted as a single standardized autoantibody assay: substrates, laboratory techniques, titre definitions and specificity varied across studies. The inference drawn was that these syndromes are autoimmune: injury exposes sequestered cardiac antigen, antibody follows, inflammation ensues. This was biologically plausible and consistent with the observations available at the time, but the studies did not establish that the antibodies themselves were pathogenic or that adaptive autoimmunity was the sole causal mechanism.

A related consequence was terminological overlap. Because the mechanism was assumed to be shared, the labels were sometimes used interchangeably, and the eponym “Dressler syndrome” came to be applied to at least three distinct referents: Dressler’s original late, febrile, relapsing post-infarction illness; any pericarditis after infarction, including the early epistenocardiac reaction that appears within days over a transmural infarct; and, loosely, pericarditis after cardiac injury of any description. In this review, we reserve “Dressler syndrome” for the delayed post-myocardial-infarction phenotype and use “post-cardiac injury syndrome” (PCIS) only as the broader trigger-defined category. The 2015 European Society of Cardiology guidelines formalised post-cardiac injury syndrome as an umbrella covering post-myocardial infarction syndrome, post-pericardiotomy syndrome and post-traumatic pericarditis, with a shared set of criteria (25). The 2025 European guidelines went further, offering inflammatory myopericardial syndrome as a provisional designation for the diagnostic interval (26), while the 2025 American College of Cardiology guidance graded pericarditis itself as definite, possible or unlikely (27).

This aggregation was clinically practical but complicates epidemiological interpretation. Early infarct-associated pericarditis and late post-myocardial-infarction pericarditis (Dressler syndrome) differ in timing, in their relation to transmurality, in recurrence risk and potentially in mechanism. Pooling them, or pooling either with post-pericardiotomy syndrome, generates incidence estimates that should not be directly compared unless case definitions, ascertainment methods, procedural settings and follow-up are sufficiently similar. **Table 1** is offered to clarify these distinctions rather than to propose a new formal taxonomy.

**Table 1:** Nosology of the post-cardiac injury pericardial syndromes: what each term denotes, what it is commonly confused with, and recommended usage.

Term	Denotes	Commonly conflated with	Recommended usage
Dressler syndrome	Late (typically 1–8 weeks), febrile, often relapsing pleuropericardial illness after myocardial infarction, with systemic inflammation	Early epistenocardiac pericarditis; any post-infarction pericardial rub or effusion	Retain as the eponym for the late post-infarction phenotype only. Do not use as a synonym for PCIS
Early infarct-associated (epistenocardiac) pericarditis	Localised pericardial inflammation over a transmural infarct within roughly 1–5 days; low recurrence risk	Dressler syndrome; post-infarction angina	Report separately in all cohorts; different timing, mechanism and prognosis
Post-pericardiotomy syndrome	PCIS following surgical opening of the pericardium	Postoperative pleural effusion; postoperative atrial fibrillation	The best-evidenced subtype and the only one with randomised prevention data
Post-cardiac injury syndrome (PCIS)	Umbrella term for the pericardial reaction to sterile cardiac injury of any cause (infarction, surgery, catheter, trauma)	Used interchangeably with each of its own subtypes	Use as the parent category; always state the trigger and latency
Inflammatory myopericardial syndrome	Provisional designation for the myocardial– pericardial inflammatory continuum before final diagnosis	Myocarditis; myopericarditis; perimyocarditis	Useful for triage; not a substitute for an aetiological diagnosis
Effusive-constrictive and transient constrictive pericarditis	Physiological complications that may follow any PCIS subtype	Established chronic constriction	Distinguish explicitly: transient constriction may resolve with anti-inflammatory therapy alone

PCIS, post-cardiac injury syndrome.

## 3 The pericardium is not a bag

Any modern account of this syndrome should begin by reconsidering the traditional view of the pericardium as a largely passive fibrous sac containing a small volume of lubricating fluid. Contemporary experimental and human data instead support the concept of the pericardium as an immunologically active serosal compartment.

The serosal mesothelium is a sentinel epithelium. It expresses pattern-recognition receptors and inflammasome machinery, and in human pericardial specimens obtained during flares of chronic pericarditis, NLRP3, ASC and caspase-1 are all more intensely expressed than in controls (19) (Level B). These observations support a role for the mesothelium as an active participant in local inflammatory signalling, although they have not been replicated in PCIS tissue.

Experimental studies further suggest that serous cavities contain resident immune populations capable of responding rapidly to adjacent tissue injury (28), and the pericardial cavity is no exception. Deniset and colleagues identified GATA6-expressing pericardial cavity macrophages which, after experimental infarction, cross into the epicardium, lose GATA6 expression and continue to exert anti-fibrotic functions; disrupting the cavity accelerated maladaptive remodelling, and equivalent cells were recoverable from human pericardial fluid (29) (Level C). These findings raised the possibility that resident pericardial macrophages contribute to reparative responses after myocardial injury and that disruption of the pericardial compartment could alter this response. However, this interpretation remains contested. A subsequent genetic lineage-tracing study found neither migration into the myocardium nor an effect on fibrosis (30), and the disagreement is unresolved. Accordingly, the available evidence establishes that the pericardial cavity is immunologically populated, but does not yet establish that depletion or displacement of GATA6-expressing macrophages causally promotes post-cardiac injury inflammation or adverse remodelling in humans.

The tissue immediately outside the cavity is similarly active. In murine infarction, pericardial adipose tissue regulates granulopoiesis, fibrosis and cardiac function after infarction, and contains lymphoid aggregates including interleukin-10–producing B cells that expand after ischaemic injury (31) (Level C). **Table 2** summarises the compartment and **Figure 1** depicts it.

**Table 2:** Constituents of the pericardial immune compartment and their putative relevance to post-cardiac injury pericarditis.

Constituent	Established function	Relevance to the syndrome	Status of the evidence
Serosal mesothelial cells	Pattern-recognition receptor expression; NLRP3, ASC and caspase-1 machinery; fluid homeostasis	Probable initiating cell type; site of inflammasome activation demonstrated in human pericarditis specimens	Human histology plus murine model; reasonably secure (Level B)
GATA6^+^ pericardial cavity macrophages	Resident, self-renewing cavity population; non-vascular access to the epicardium	Anti-fibrotic after experimental infarction; cavity disruption accelerated adverse remodelling; present in human pericardial fluid	Contested — lineage tracing has not reproduced migration or the fibrosis phenotype. (Level C)
Pericardial and epicardial adipose tissue	Regulates granulopoiesis and post-infarction fibrosis; hosts lymphoid aggregates	Local source of leucocyte supply and of IL-10-producing B cells after infarction	Murine with supporting human tissue observations(Level C)
Pericardial fluid (15–35 mL)	Ultrafiltrate plus locally secreted mediators and cells	Accessible compartment for cytokine profiling; routinely aspirated and routinely discarded	Almost entirely unexploited in PCIS research
Recruited neutrophils and monocyte-derived macrophages	Effectors of the acute exudative response	Substrate for effusion, tamponade, organisation and constrictive fibrosis	Classical pathology; well described, poorly quantified in PCIS specifically

ASC, apoptosis-associated speck-like protein containing a CARD; IL, interleukin; NLRP3, NACHT, leucine-rich repeat and pyrin domain-containing protein 3; PCIS, post-cardiac injury syndrome.

**Figure 1:**
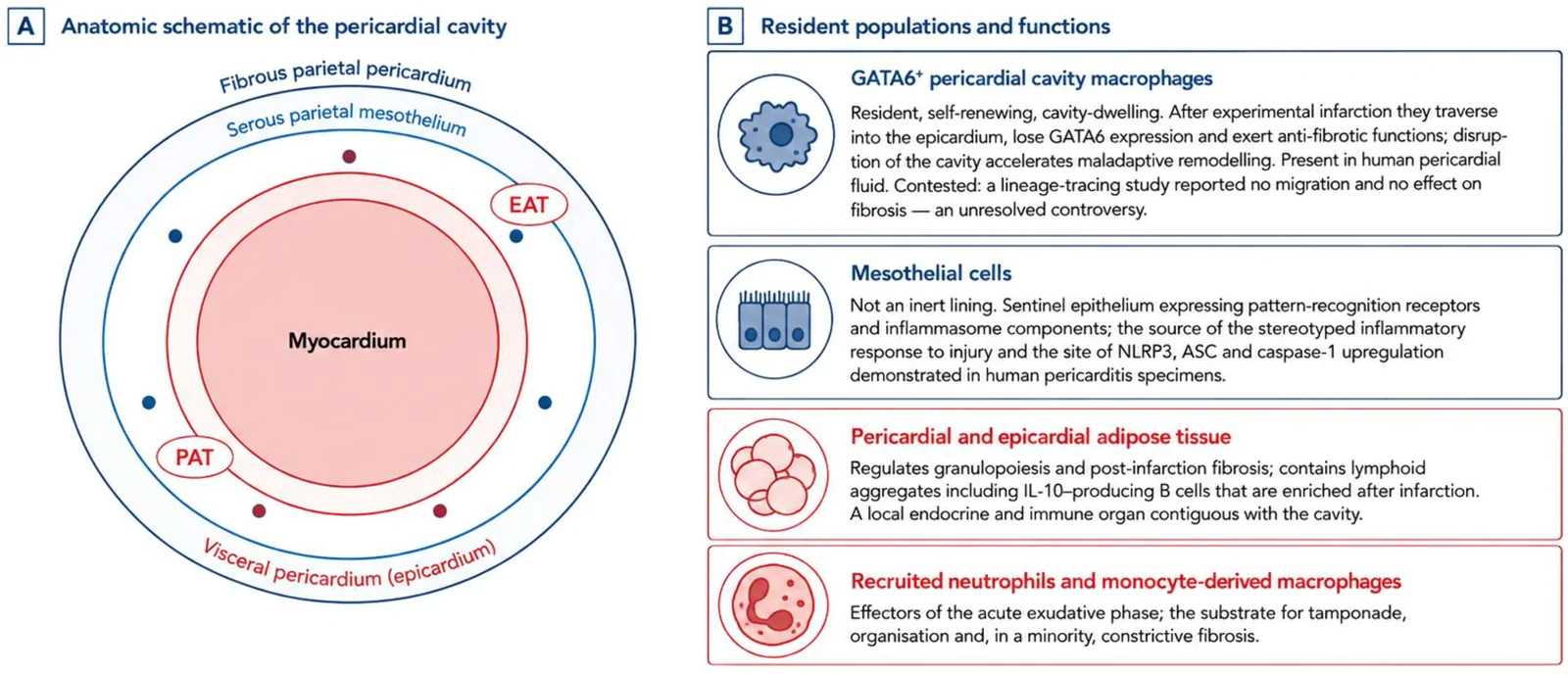
The pericardial cavity as an immunological compartment: cellular architecture and functional implications in cardiac injury. **(A)** Anatomic organization of the pericardial cavity. The pericardial cavity is bounded by the fibrous parietal pericardium, serous parietal mesothelium, and visceral pericardium (epicardium) surrounding the myocardium. The cavity contains resident immune cells and is anatomically contiguous with epicardial adipose tissue (EAT) and pericardial adipose tissue (PAT), forming an integrated cardio-immunologic niche. **(B)** Major resident cellular populations and their functions. GATA6^+^ cavity-resident macrophages constitute a self-renewing population with reparative and anti-fibrotic properties that can migrate to the injured epicardium after myocardial infarction. Mesothelial cells actively regulate innate immune sensing through pattern-recognition receptors and inflammasome signalling rather than serving as a passive lining. Epicardial and pericardial adipose tissues function as local immunometabolic organs, supporting leukocyte homeostasis and post-infarction repair through cytokine production and lymphoid aggregates. During acute pericardial inflammation, recruited neutrophils and monocyte-derived macrophages dominate the inflammatory exudate, contributing to effusion formation, organization, and, in some cases, constrictive fibrosis (Level B-C). (Proposed mechanism underlying post-cardiac injury syndrome. Interventions that disrupt the pericardial cavity—including pericardiotomy, pericardiocentesis, catheter ablation, or lead perforation—may not simply expose cardiac tissues to damage-associated molecular signals but may also deplete or perturb resident immunoregulatory populations that normally restrain inflammation and fibrosis. This conceptual model suggests that loss of pericardial immune homeostasis may contribute to post-cardiac injury syndrome and adverse pericardial remodelling, although direct evidence in humans remains limited.). EAT, epicardial adipose tissue; PAT, pericardial adipose tissue; GATA6, GATA-binding protein 6.

These observations generate a clinically relevant, testable but currently unproven hypothesis: Pericardiotomy, pericardial drainage, ablation-related injury, lead perforation, and other interventions may perturb resident immune populations and local immunoregulatory networks to differing degrees (Level C). If some of these resident populations exert net restraining or reparative effects, their disruption could theoretically lower the threshold for a subsequent inflammatory response to cardiac injury. This possibility is particularly relevant to post-pericardiotomy syndrome, but direct human evidence that loss or redistribution of resident pericardial immune cells contributes causally to the syndrome is currently lacking. The concept should therefore be regarded as a testable mechanistic hypothesis rather than an established explanation for the high incidence of post-pericardiotomy inflammation. Prospective human studies incorporating serial pericardial fluid or tissue sampling before and after defined cardiac injury would be required to determine whether perturbation of resident immunoregulatory populations precedes, accompanies, or predicts the development of post-cardiac injury syndrome.

## 4 Innate and adaptive immunity: from an autoimmune to an integrated model

A distinction runs through this section and should be stated at the outset. Randomised trials in recurrent pericarditis show that interleukin-1 blockade prevents recurrence in selected patients; that is evidence of therapeutic efficacy in one phenotype (Level B). It does not establish that late post-infarction Dressler syndrome depends mechanistically on interleukin-1 to the same degree, because the two conditions differ in trigger, in the presence of a healing myocardial wound, and in concomitant antithrombotic therapy. No study has measured inflammasome activation or interleukin-1 activity in pericardial tissue or fluid from patients with late post-infarction pericarditis. The innate immune interpretation developed below should therefore be read as a testable framework, not as a mechanistic reclassification of the syndrome.

### 4.1 The adaptive account, and why it no longer carries the weight placed on it alone

The autoimmune model holds that sterile necrosis exposes sequestered intracellular antigens — actin, myosin, sarcolemmal constituents — which prime a humoral response; antibody and immune complexes then deposit in the pericardium and drive inflammation. It has real explanatory virtues. It offers one plausible explanation for latency, because adaptive immune responses require time to develop, and for recurrence, because immunological memory may sustain susceptibility to repeated inflammatory episodes. It is also compatible with corticosteroid responsiveness, although this is not specific evidence for an antibody-mediated mechanism.

The difficulties are, on inspection, serious. Heart-reactive antibody was found in essentially all patients with post-pericardiotomy syndrome, but transient low-titre antibody also appeared in a large fraction of postoperative patients who remained entirely well (22, 23), so titre discriminates poorly at the level of the individual patient. Moreover, the historical term “heart-reactive antibody” encompasses heterogeneous antibody signals measured with era-specific assays and should not be interpreted as a single standardized autoantibody biomarker. No passive transfer experiment has established causal sufficiency in this setting. The most striking finding in Engle’s prospective paediatric work was not antibody alone but its co-occurrence with a rise in antiviral antibody in 68 per cent of affected children against 5 per cent of unaffected ones (24) — interpreted then as viral reactivation, but also compatible with the possibility that a concurrent innate inflammatory stimulus contributed to disease expression.

Therapeutic observations also argue against a purely antibody-driven model, although they do not exclude an important contribution from adaptive immunity. Corticosteroids control the flare and then permit it to return; high initial doses are associated with a more complicated course, and lower doses with slower tapers do better (32, 33). Azathioprine and intravenous immunoglobulin remain third-line and evidence-poor. By contrast, IL-1-directed therapies have shown substantial efficacy in recurrent pericarditis (Level B). These therapeutic data therefore weaken the concept of PCIS as an exclusively antibody-mediated disorder, but they do not establish that adaptive immune mechanisms are non-causal. A more plausible interpretation is that innate and adaptive pathways may contribute to different phases or phenotypes of the syndrome. Two errors should be avoided in interpreting this literature. The first, historical, is to treat heart-reactive antibody as proof of an antibody-mediated disease. The second, more contemporary, is to infer from the failure of that proof that adaptive immunity is epiphenomenal: absence of evidence of pathogenicity is not evidence of irrelevance. The latency of PCIS and its tendency to recur are both compatible with a contribution from antigen priming and immunological memory. The formulation most consistent with current evidence is that sterile injury initiates innate immune activation, while adaptive responses may contribute to amplification, persistence or recurrence in susceptible individuals. This is the formulation adopted throughout this review.

### 4.2 The innate immune account

In idiopathic and recurrent pericarditis, the inflammatory response of the pericardial mesothelium appears to depend substantially on NLRP3 inflammasome activation and interleukin-1 signalling (Level B) (34, 35); whether the same dependency holds in late post-infarction pericarditis has not been tested. Assembly of the inflammasome cleaves pro-interleukin-1β to its mature form; interleukin-1α, released constitutively from necrotic cells independently of the inflammasome, engages the same receptor in parallel and has been shown to be a crucial proximal danger signal in acute myocardial infarction (36) (Level C). Interleukin-1 receptor engagement then drives nuclear factor-κB–dependent transcription of pro-interleukin-1β, providing a potential positive-feedback mechanism through which inflammation may persist beyond the initial tissue injury and contribute to recurrence. Whether this pathway alone accounts for the characteristic latency or recurrence of PCIS, however, remains uncertain.

Support for this pathway derives from several complementary but distinct levels of evidence. Intrapericardial zymosan A in mice reproduces effusion, pericardial thickening and interleukin-1α and interleukin-1β upregulation through an NLRP3-dependent mechanism, and the phenotype responds to colchicine, to a selective NLRP3 inhibitor, to anakinra and to an interleukin-1 trap (Level C); the same inflammasome components are upregulated in human pericardium (19) (Level B).

In the clinic, colchicine reduced incessant or recurrent pericarditis from 37.5 to 16.7 per cent in ICAP (15) and reduced recurrence in the broader recurrent-pericarditis populations studied in CORP and CORP-2 (14, 16) (Level B). Direct evidence in a trigger-defined PCIS population comes from COPPS and COPPS-2, in which colchicine reduced post-pericardiotomy syndrome – COPPS-2 from 29.4 to 19.4 per cent, a number needed to treat of ten (17, 18, 37) (Level A). In AIRTRIP, a randomised withdrawal design in colchicine-resistant, corticosteroid-dependent disease, recurrence occurred in nine of ten patients on placebo and two of eleven on anakinra (11). In RHAPSODY, rilonacept produced so few events that median time to first adjudicated recurrence could not be estimated, against 8.6 weeks on placebo, with a hazard ratio of 0.04 (12). Long-term extension confirmed durability and recurrence on withdrawal (38); the IRAP registry replicated the anakinra effect in real-world practice (39); goflikicept has since delivered comparable results (40). Collectively, these studies provide strong pharmacological evidence that interleukin-1 signalling is clinically important in recurrent pericarditis (Level B). None enrolled a PCIS population.

### 4.3 Host susceptibility: the monogenic shadow

The third strand is genetic, and it is the one cardiologists most often overlook. Colchicine and interleukin-1 blockade did not arrive in pericardiology from cardiovascular research; they were imported from familial Mediterranean fever and the cryopyrinopathies, and their efficacy in recurrent pericarditis is consistent with, although does not by itself establish, an important role for innate immune and inflammasome-mediated pathways. Peet and colleagues sequenced MEFV, MVK, NLRP3 and TNFRSF1A in 128 patients with idiopathic recurrent pericarditis at a national autoinflammatory referral centre and found rare deleterious MEFV variants significantly more frequent than in ancestry-matched controls; pericarditis occurred across every monogenic autoinflammatory disease examined, corticosteroid dependence affected 28.7 per cent of the pericarditis cohort and was associated with chronic pain, C-reactive protein was elevated in 89.0 per cent and extrapericardial effusions were present in half (41). Subsequent series have reported comparable variant enrichment (42) (Level B). A polygenic susceptibility lowering the threshold for recurrent inflammation has been proposed (43, 44). Whether an analogous genetic predisposition modifies the risk of post-injury pericarditis is plausible but remains unproven. In PCIS, a defined cardiac injury could provide the initiating inflammatory stimulus in a genetically susceptible host; however, direct genetic studies specifically in Dressler syndrome and other trigger-defined PCIS populations are required before this mechanism can be inferred.

## 5 A pericardial injury-threshold model

Contemporary integrated accounts of PCIS already incorporate sterile injury, damage-associated molecular pattern release, inflammasome activation, interleukin-1 signalling and host susceptibility (7, 45). We do not claim novelty for recognising these components. The model proposed here adds four elements. First, it treats injury magnitude (necrotic mass) and the directness of pericardial and mesothelial exposure as separate inputs. This offers a testable explanation for why procedures producing little necrosis, such as catheter ablation, can carry a higher observed pericarditis risk than reperfused infarction. Second, it treats procedural perturbation of the resident pericardial immune compartment as a modifier of the threshold, a hypothesis currently supported only at Level C. Third, it assigns adaptive immunity a defined and testable role as a potential amplifier of persistence and recurrence, rather than either the initiator or an epiphenomenon. Fourth, it is paired with specified falsifiable predictions and discriminating experiments (**Table 3**). One of these is that ultra-early pericardial fluid interleukin-1 kinetics after ablation should precede any adaptive response. The value of the model lies in these testable distinctions rather than in its components.

**Table 3:** Comparative appraisal of the autoimmune and autoinflammatory models, with the experiment that would discriminate between them.

Domain	Adaptive contribution	Innate contribution	Discriminating evidence or experiment
Proposed role	Amplifier of persistence and recurrence (proposed)	Initiator via DAMP-driven NLRP3 activation and IL-1α (proposed)	Serial pericardial fluid cytokine profiling versus antibody titre, referenced to clinical activity
Latency	Time to mount a humoral response	Time for a proposed IL-1 amplification loop to reach symptomatic amplitude	Ultra-early sampling within 72 hours of ablation, before any adaptive response is possible
Recurrence	Immunological memory	IL-1 auto-induction persisting after the initiating stimulus resolves	Recurrence on withdrawal of IL-1 blockade despite unchanged antibody titre
Therapeutic response	Corticosteroids, azathioprine, IVIG (evidence-poor)	Colchicine; IL-1 blockade; direct NLRP3 inhibition (investigational)	Recurrent pericarditis (Level B): marked reduction in recurrence with IL-1 blockade in randomised-withdrawal trials of responders; no PCIS data. NLRP3 versus IL-1 blockade would separate inflammasome from IL-1α-driven disease
Genetic architecture	HLA and adaptive-immunity associations expected	Inflammasome-pathway variants (MEFV, NLRP3, TNFRSF1A) expected	Demonstrated for idiopathic recurrent pericarditis (Level B); untested in PCIS
Current interpretation	Associative Level A evidence (historical assays); causal role unresolved	Supported at Levels B and C; untested in late post-infarction PCIS	The models are complementary rather than exclusive; the experiments above would establish their relative contributions

CRP, C-reactive protein; DAMP, damage-associated molecular pattern; HLA, human leucocyte antigen; IL, interleukin; IVIG, intravenous immunoglobulin; LGE, late gadolinium enhancement; NLRP3, NACHT, leucine-rich repeat and pyrin domain-containing protein 3; PCIS, post-cardiac injury syndrome.

These strands can be integrated into a single, deliberately simple proposition. The probability that a given cardiac injury produces clinical pericarditis may be conceptualised as depending on whether several interacting determinants collectively cross an inflammatory threshold (**Figure 2**).

**Figure 2:**
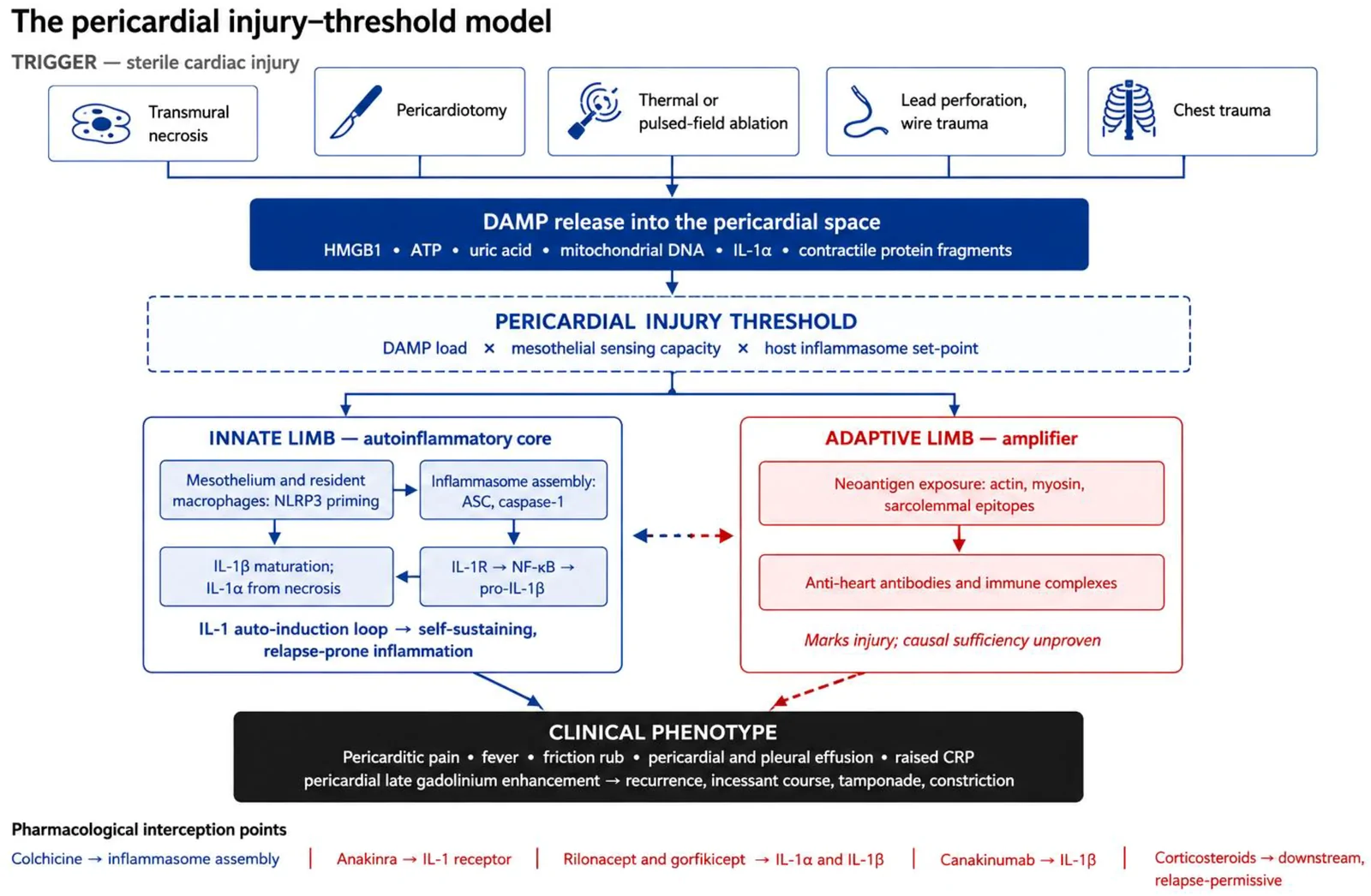
The pericardial injury-threshold model. Sterile cardiac injury of any aetiology releases damage-associated molecular patterns into the pericardial space; whether clinical inflammation follows depends on a threshold set by the product of antigen and DAMP load, mesothelial sensing capacity and host inflammasome set-point. Above threshold, mesothelial cells and resident macrophages assemble the NLRP3 inflammasome, and interleukin-1β together with necrosis-derived interleukin-1α establishes a self-sustaining loop that accounts for the relapsing natural history. The adaptive limb amplifies and marks, but has not been shown to initiate. ASC, apoptosis-associated speck-like protein containing a CARD; CRP, C-reactive protein; DAMP, damage-associated molecular pattern; HMGB1, high mobility group box 1; IL, interleukin; IL-1R, interleukin-1 receptor; NF-κB, nuclear factor-κB; NLRP3, NACHT, leucine-rich repeat and pyrin domain-containing protein 3.

The first is damage-associated molecular pattern load — the quantity of HMGB1, ATP, uric acid, mitochondrial DNA, interleukin-1α and contractile protein fragments delivered into the pericardial space. This scales with necrotic mass, but crucially it also scales with the directness of pericardial contact, which is not the same variable. The second is mesothelial sensing capacity: the density and priming state of mesothelial cells and resident macrophages, modified by thermal, cryogenic or electroporative injury to the mesothelium itself, and potentially by the surgical evacuation of the compartment discussed above. The third is the host inflammasome set-point — a constitutional parameter shaped by variants in MEFV, NLRP3 and related genes (41, 42), by age, and by concurrent innate stimuli. The adaptive humoral limb may operate downstream or in parallel with these innate processes. It is proposed as a potential amplifier of persistence and recurrence, whose causal contribution remains unresolved.

The attraction of this formulation is that it provides a possible framework for reconciling apparently discordant epidemiological observations. Reperfusion lowers the first input, which may help explain why late post-myocardial-infarction pericarditis became less frequent in the reperfusion era, without requiring a change in underlying host immune susceptibility. Catheter ablation delivers a relatively limited necrotic mass but can injure the mesothelium directly and repeatedly, potentially increasing pericardial innate immune activation. This framework is compatible with the observation that radiofrequency ablation has been associated with a higher observed risk of post-ablation pericarditis than cryoballoon ablation (7, 10), and with reports of the syndrome even after comparatively limited lesion sets, such as slow-pathway ablation for atrioventricular nodal re-entrant tachycardia (46). Pericardiotomy may simultaneously increase tissue-derived inflammatory signals, directly disrupt the mesothelial compartment, and alter resident immunoregulatory cell populations. These mechanisms provide a testable explanation for the comparatively high incidence of post-pericardiotomy syndrome, but direct human evidence establishing this causal pathway remains limited. The steep age gradient in Engle’s paediatric data, with 3.5 per cent incidence below two years against 27 per cent overall, is compatible with, but does not establish, age-dependent variation in host inflammatory susceptibility (24).

A model of this kind earns its place only if it can be wrong. It generates four hypotheses that current technology could examine. Pericardial fluid interleukin-1α and interleukin-1β, sampled serially, may track clinical activity more closely than anti-heart antibody titre. Lesion characteristics reflecting mesothelial contact — posterior wall involvement, extravenous lesions, energy modality — may prove more informative than total ablated area in predicting post-ablation pericarditis. Gain-of-function inflammasome variants may be enriched in recurrent relative to single-episode post-cardiac injury pericarditis. And residual cases of classical Dressler syndrome in the reperfusion era may be preferentially observed among patients with a larger injury burden or greater host inflammatory susceptibility, rather than being uniformly distributed across the infarct population. These predictions require prospective testing. **Table 3** sets out the comparative appraisal in full.

## 6 Epidemiology: changing clinical settings in recognition

Before reperfusion, clinically evident pericardial involvement after infarction was reported in seven to twenty-three per cent of patients, with higher rates at autopsy, and the late Dressler phenotype in three to five per cent (3, 7). The decline was then documented serially: Lichstein and colleagues found no late syndrome in a 1980 coronary care cohort despite early pericarditis in 6.4 per cent (3), Shahar and colleagues described its disappearance in the thrombolytic era (4), and contemporary estimates place the early and late forms below five and below one per cent respectively (7).

Three explanations may contribute to this decline, although their relative importance remains uncertain and they are not mutually exclusive. The conventional one is biological: earlier and more complete reperfusion may reduce necrotic mass and with it the damage-signal load presented to the pericardium (5, 7). The second is pharmacological. Angiotensin-converting enzyme inhibitors, beta-blockers and statins became standard over a similar period, and each has potentially relevant anti-inflammatory or immunomodulatory effects (5). Low-dose colchicine, now supported for secondary prevention after infarction and in chronic coronary disease (47, 48), is particularly relevant because it also reduces recurrence in established pericarditis, although this does not establish that its contemporary cardiovascular use contributed materially to the historical decline in Dressler syndrome. The third possibility is ascertainment-related: part of the reported decline predated modern reperfusion, raising the possibility that changes in clinical recognition, diagnostic practices, coding, or follow-up also contributed.

Discriminating between these explanations matters because they imply different residual burdens, particularly where reperfusion is delayed or unavailable.

Set against the decline is the recognition of PCIS across an expanding range of procedures (**Table 4**). Taken together, these data indicate that the clinical settings in which PCIS is encountered and recognised have changed. They do not show that the same biological disease burden has migrated from infarction to the procedural laboratory, because the underlying studies differ in population, era, diagnostic criteria, surveillance intensity and denominator. Post-pericardiotomy syndrome has been reported in fifteen to thirty per cent of cardiac operations, with risk varying by operation type and with postoperative mortality reported to be higher among affected patients in a nationwide cohort (8, 49, 50). Acute pericarditis has been reported in 3.7 per cent of patients after atrial fibrillation ablation in one national analysis, with radiofrequency energy and younger age associated with a higher observed risk, and in as many as 10.2 per cent in a retrospective cohort of 2,215 patients when suspected cases were counted (7, 10, 51). Iatrogenic pericarditis after percutaneous coronary intervention, lead insertion and other catheter-based work has been reported at frequencies of approximately 0.5 to 5 per cent (7). The syndrome has been described after pulsed-field ablation (52), and constrictive pericarditis after catheter ablation, though rare, is documented (53).

**Table 4:** Triggers, latency and reported burden of post-cardiac injury pericarditis in contemporary practice.

Trigger	Typical latency	Reported incidence	Distinguishing features	Quality of the evidence
Late post-myocardial infarction (Dressler syndrome)	1–8 weeks; occasionally to 3 months	<1% after primary PCI; 3–5% before reperfusion	Fever, relapse, pleural involvement, marked CRP rise; associated with large, late or failed reperfusion	Case reports and historical cohorts; no prospective modern series exists
Early infarct-associated (epistenocardiac) pericarditis	1–5 days	<5% currently; 7–23% historically	Localised over transmural infarct; low recurrence risk; delayed angioplasty the main risk factor	Observational; consistently reported, rarely adjudicated
Post-pericardiotomy syndrome	1–6 weeks	15–30%, varying by operation type	Commonest subtype; pleural effusion frequent; associated with postoperative mortality	Strongest evidence base: two randomised prevention trials and a nationwide cohort
Atrial fibrillation catheter ablation	Hours to 4 weeks	3.7% adjudicated; to 10.2% including suspected cases	Radiofrequency energy and younger age independent predictors; posterior wall and extravenous lesions implicated	Single-centre and registry cohorts; definitions not harmonised
Device implantation, lead perforation, PCI, structural intervention	Days to weeks	0.5–5%, procedure dependent	Frequently attributed to procedural discomfort and under-recognised; tamponade may be the presenting event	Case reports and complication registries; no dedicated studies
Blunt or penetrating chest trauma	Days to weeks	Not reliably quantified	Confounded by haemopericardium and concomitant injury	Case series only

CRP, C-reactive protein; PCI, percutaneous coronary intervention.

Prognosis also argues against assuming that all post-cardiac injury pericardial syndromes are uniformly benign. In a prospective cohort of 500 patients with a first episode of acute pericarditis followed for a median of 72 months, constrictive pericarditis developed in 0.48 per cent of those with idiopathic or viral disease but in 8.3 per cent of those with a non-idiopathic aetiology, an incidence rate of 4.40 per 1,000 person-years in the connective tissue disease and pericardial injury syndrome stratum — roughly six times the idiopathic rate (54). This stratum was not PCIS-specific, but injury-associated forms should not be presumed benign.

## 7 Phenotype and natural history

The presentation Dressler recorded has not altered. Pleuritic, positional chest pain — worse supine, eased by sitting forward — is the cardinal symptom, and it is the postural and respiratory dependence rather than the character of the pain that separates it from recurrent ischaemia. Fever without an alternative explanation is characteristic; Dressler himself drew attention to a distinctive temperature curve, low-grade with peaks coinciding with exacerbations of pain (1, 2). A friction rub is highly specific, audible in a minority, and evanescent enough that its absence at one examination means little. Pericardial effusion is usually small to moderate, and pleural effusion, often left-sided, is common enough to have become a diagnostic criterion. Systemic inflammatory markers, including C-reactive protein and the erythrocyte sedimentation rate, are frequently elevated, often with leucocytosis; however, these findings are neither specific for Dressler syndrome nor reliably distinguishable from the inflammatory response accompanying recent myocardial infarction or other post-injury states.

The longer-term clinical course is less well characterised specifically for contemporary Dressler syndrome, and much of the available evidence regarding recurrence and complications derives from broader populations with acute or recurrent pericarditis. Complications or recurrence affect up to thirty per cent of patients with acute pericarditis of any cause (34). Corticosteroid dependence has been reported in recurrent pericarditis cohorts and represents an important treatment-related concern; in one large referral cohort, it was present in 28.7 per cent of patients and was associated with chronic pain (41). Effusive-constrictive and transient constrictive physiology may supervene, and the latter deserves emphasis because it can resolve with anti-inflammatory therapy alone — a distinction that may help avoid premature referral for pericardiectomy. Established constriction in non-idiopathic disease occurs at roughly six times the idiopathic rate (54), recurrent and hospitalized pericarditis also carry substantial morbidity (55, 56), although none of these estimates is Dressler-specific.

## 8 Diagnosis

Post-cardiac injury syndrome is diagnosed when at least two of five features follow documented cardiac injury: fever without alternative cause; pericarditic or pleuritic pain; pericardial or pleural rub; new or worsening pericardial effusion; and pleural effusion with elevated C-reactive protein (25). These criteria are expert consensus. To our knowledge, they have not been prospectively validated in a dedicated post-cardiac injury population, and their diagnostic operating characteristics remain uncertain. Moreover, they were adapted largely from the clinical framework used for acute pericarditis rather than derived and validated specifically after cardiac injury (7). A dedicated proposal for post-pericardiotomy criteria exists and could be adapted, but we identified no prospective validation establishing its diagnostic performance (57). **Table 5** compares the available frameworks.

**Table 5:** Diagnostic frameworks applicable to Dressler syndrome and post-cardiac injury syndrome.

Framework	Construct	Strengths	What it leaves undefined
Dressler, 1956–1959	Clinical gestalt: fever, pleuropericardial pain, rub, effusion, leucocytosis, relapsing course after infarction	Captured the defining temporal and relapsing character	No thresholds, no exclusion rules, no separation from early infarct-associated pericarditis
ESC 2015 PCIS criteria	≥2 of 5: fever without alternative cause; pericarditic or pleuritic pain; pericardial or pleural rub; new or worsening pericardial effusion; pleural effusion with raised CRP	First operational definition; unified the post-infarction, surgical and traumatic subtypes	Never prospectively validated; no required latency window; no imaging tier; no activity grading
ESC 2025	Inflammatory myopericardial syndrome as a provisional umbrella; presentation-driven pathways; expanded role for CMR	Resolves the myocarditis– pericarditis overlap; integrates tissue characterisation	PCIS remains subordinate; procedural triggers not separately operationalised
ACC 2025 concise clinical guidance	Definite, possible and unlikely pericarditis by typical pain plus criterion count; proposed pericardial LGE grading	First US consensus; explicitly phenotype-driven and imaging-anchored	Tiers unvalidated in a post-injury population; access to CMR assumed
Candidate research definition requiring prospective validation (this review; not a clinical definition)	Tier 1 (clinical): ≥2 of 5 ESC criteria plus objective CRP elevation interpreted against expected post-injury kinetics, with onset 7 days to 3 months after myocardial infarction or 72 hours to 3 months after surgical, catheter or traumatic injury. Tier 2 (research confirmation): Tier 1 plus pericardial LGE or oedema on CMR	Separates ascertainment from confirmation; deployable in registries without universal access to CMR	Requires prospective validation against blinded adjudication with CMR as an index test — the first work package of the programme proposed in **Table 8**

ACC, American College of Cardiology; CMR, cardiovascular magnetic resonance; CRP, C-reactive protein; ESC, European Society of Cardiology; LGE, late gadolinium enhancement; PCIS, post-cardiac injury syndrome.

Two practical problems follow. The first is that the criteria may be least discriminating in clinical settings in which diagnostic uncertainty carries the greatest consequences. In the weeks after infarction, pericarditic pain competes with recurrent ischaemia, stent thrombosis, free-wall rupture and pulmonary embolism; the electrocardiogram is confounded by evolving infarct patterns; and a small effusion may reflect the antecedent myocardial injury rather than PCIS itself. After ablation, procedure-related chest discomfort and inflammatory changes may similarly reduce the specificity of individual diagnostic features. The second is that fever and effusion are shared with infection, which must be excluded before intensifying anti-inflammatory treatment. This is why the pathway in **Figure 3** places exclusion of the time-critical mimics ahead of criterion-counting, rather than treating diagnosis as a scoring exercise.

**Figure 3:**
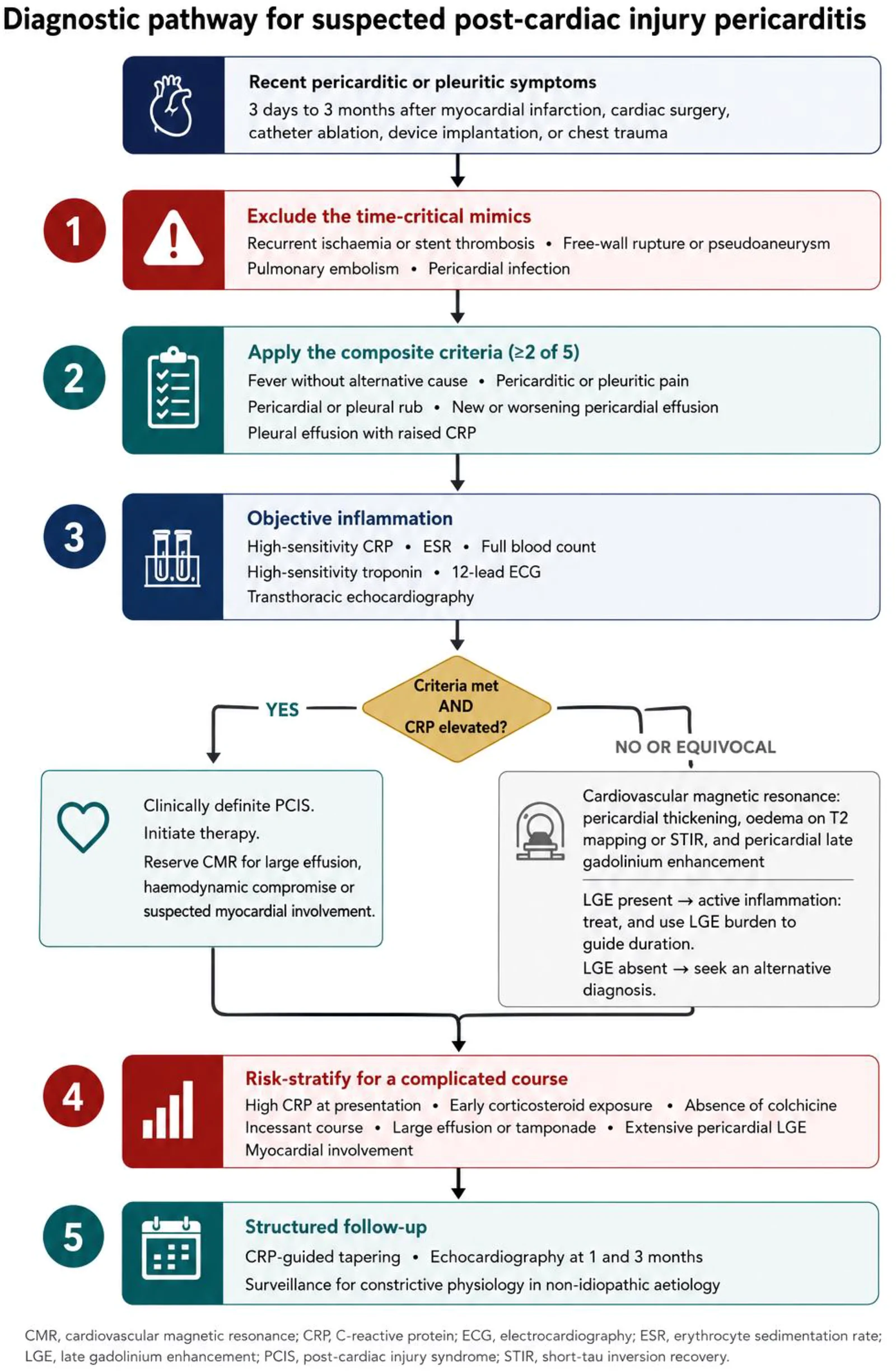
Proposed diagnostic pathway for clinical use within current guideline criteria. Exclusion of time-critical mimics precedes criterion-counting, because the clinical features of the syndrome overlap substantially with recurrent ischaemia, mechanical complication, pulmonary embolism and pericardial infection in the post-injury window. Cardiovascular magnetic resonance is positioned as arbiter for equivocal presentations rather than as a routine first study. Absence of late gadolinium enhancement should be interpreted in light of timing and prior anti-inflammatory therapy, which may attenuate enhancement. The trigger-specific time windows follow the candidate research definition in **Table 5** and require prospective validation. CMR, cardiovascular magnetic resonance; CRP, C-reactive protein; ESR, erythrocyte sedimentation rate; LGE, late gadolinium enhancement; PCIS, post-cardiac injury syndrome; STIR, short-tau inversion recovery.

There is no validated syndrome-specific diagnostic biomarker. C-reactive protein is the workhorse and is genuinely useful — elevated in the great majority, correlated with activity, and the basis of tapering in practice — but it is entirely non-specific and rises after infarction and cardiac surgery irrespective of the pericardium. Its interpretation is therefore strongly dependent on timing, baseline post-injury inflammatory kinetics, and prior anti-inflammatory treatment; a normal value may be less informative after delayed presentation or treatment initiation, whereas an elevated value early after infarction or surgery may not discriminate PCIS from the expected inflammatory response. Anti-heart antibody testing is unstandardised, largely unavailable and, as argued above, poorly discriminating. High-sensitivity troponin identifies myocardial involvement, which matters prognostically (58), but does not identify the syndrome.

Cardiovascular magnetic resonance has consequently assumed a role the criteria do not formally acknowledge. Pericardial thickening, oedema on T2-weighted imaging or T2 mapping, and pericardial late gadolinium enhancement together can provide objective evidence of active pericardial inflammation and may support the diagnosis when clinical findings are equivocal (27, 59, 60). However, CMR should be regarded as a supportive or confirmatory tissue-characterisation tool rather than as an intrinsic component of the diagnostic reference standard, particularly because incorporating it into both the definition and its validation would risk circularity. Both the 2025 European guidelines and the American guidance lean more heavily on tissue characterisation (26, 27), and enhancement burden is increasingly used to inform treatment duration.

Result on imaging carries an equity consequence. If confident diagnosis depends increasingly on access to magnetic resonance, the syndrome may be more readily recognised in centres with advanced imaging than in resource-limited settings, potentially distorting observed geographic patterns of disease. Any harmonised definition must therefore specify a clinically anchored tier that does not depend on advanced imaging, alongside an imaging-confirmed tier for research use — which is the proposal we advance in the final row of **Table 5**. We regard this framework as a candidate research definition that requires prospective derivation and validation against blinded expert adjudication, with CMR evaluated as an additional diagnostic test rather than incorporated into the same reference standard.

Each element of this candidate definition is a starting value for prospective derivation, not a biologically fixed boundary. The lower time bound is trigger-specific. After myocardial infarction it is set at 7 days, to separate late (Dressler) pericarditis from early infarct-associated pericarditis, which typically occurs within the first 1–5 days and differs in mechanism and recurrence risk (**Table 1**). After surgery or catheter procedures it is set at 72 hours, to exclude the immediate post-procedural pain and pericardial irritation that accompany the injury itself. The upper bound of 3 months reflects the latency distribution reported since Dressler’s original series, in which most cases present within weeks. Extending the window further would increasingly capture idiopathic or viral pericarditis unrelated to the index injury. The requirement for C-reactive protein elevation anchors Tier 1 in objective inflammation, because each clinical criterion is individually non-specific after cardiac injury. Because C-reactive protein rises after infarction and surgery irrespective of pericardial involvement, it should be interpreted against expected post-injury kinetics: a secondary rise after an initial decline, or persistent elevation beyond the expected post-injury interval, is more informative than a single absolute value. The optimal definition is a derivation question. Tier 2 adds pericardial late gadolinium enhancement or oedema on CMR, and is intended for research confirmation, not as a requirement for clinical diagnosis. Alternative lower bounds (1 and 14 days), an upper bound of 6 months, and alternative C-reactive protein definitions should be prespecified as sensitivity analyses, as should the handling of expected post-injury effusions. Until such validation is complete, the ESC and ACC criteria remain the clinical reference.

## 9 Treatment, and the problem of the healing infarct

Evidence for treating PCIS is best read by drug class and by population, because the strength of evidence differs sharply along both axes (**Table 6**). Three differences separate late post-infarction pericarditis from the recurrent pericarditis populations in which most targeted therapies were tested: its trigger is a defined sterile injury rather than an idiopathic or viral process; it occurs while a myocardial wound is still healing; and it usually arises during dual antiplatelet therapy or anticoagulation, which alters both drug choice and the risk of haemorrhagic pericardial effusion. Each of these differences is a reason why efficacy in recurrent pericarditis cannot be assumed to transfer quantitatively, or safely, to the post-infarction setting.

**Table 6:** Hierarchy of therapeutic evidence by drug class and population, with regimen and considerations after myocardial infarction.

Drug class and regimen	Trigger-defined PCIS (Level A)	Other human pericarditis (Level B)	Late post-MI pericarditis	Current status in PCIS
Aspirin 750–1000 mg three times daily, tapered by CRP; NSAIDs	No treatment RCT	Standard background therapy in colchicine RCTs	Aspirin preferred after MI (consensus); preserves antiplatelet cover; non-aspirin NSAIDs discouraged early (experimental and observational data)	Guideline first line; aspirin preferred after MI
Colchicine 0.5 mg once or twice daily by weight; 3 months acute, 6 months recurrent	RCT evidence for prevention of post-pericardiotomy syndrome (COPPS, COPPS-2)	RCTs in acute and recurrent pericarditis (COPE, ICAP, CORP, CORP-2)	No RCT; safety of low-dose use after MI shown in COLCOT; pericarditis not reported	Best-supported PCIS-specific agent; guideline-recommended
Corticosteroids: prednisone 0.2–0.5 mg/kg, very slow taper	No RCT	Observational; high dose associated with recurrence	Concern for impaired collagen deposition and free-wall rupture during early healing (observational)	Individualised; low dose, slow taper
IL-1 blockade: anakinra 100 mg daily; rilonacept weekly after loading; goflikicept; canakinumab (anti-IL-1β, long half-life)	No RCT	Randomised-withdrawal RCTs in colchicine-resistant, inflammatory recurrent pericarditis (AIRTRIP, RHAPSODY); goflikicept phase II/III; canakinumab not studied in pericarditis	No study; anakinra tested in acute STEMI without a pericarditis endpoint; short half-life of anakinra an advantage if withdrawal is needed	Extrapolated; refractory inflammatory recurrent PCIS; investigational during infarct healing; access and cost constraints
Direct NLRP3 inhibitors (oral)	None	Open-label phase II ongoing [83]	None	Investigational

CRP, C-reactive protein; IL, interleukin; MI, myocardial infarction; NLRP3, NACHT, leucine-rich repeat and pyrin domain-containing protein 3; NSAID, non-steroidal anti-inflammatory drug; PCIS, post-cardiac injury syndrome; RCT, randomised controlled trial; STEMI, ST-elevation myocardial infarction. Trial acronyms are expanded in the reference list.

The general principles of pericardial therapy are well codified (61–65); what follows concerns the modifications imposed by recent cardiac injury, with particular attention to the distinction between guideline-supported management and therapeutic extrapolation from broader pericarditis populations. Aspirin at anti-inflammatory doses is the anchor after infarction, both because antiplatelet cover must be preserved and because non-aspirin anti-inflammatory alternatives may raise specific concerns during infarct healing. Colchicine is added for three months and extended to six for recurrence, tapered under C-reactive protein guidance. Interleukin-1 blockade is discussed below (66, 67). **Table 6** summarises the evidence hierarchy and **Figure 4** the stepped framework.

**Figure 4:**
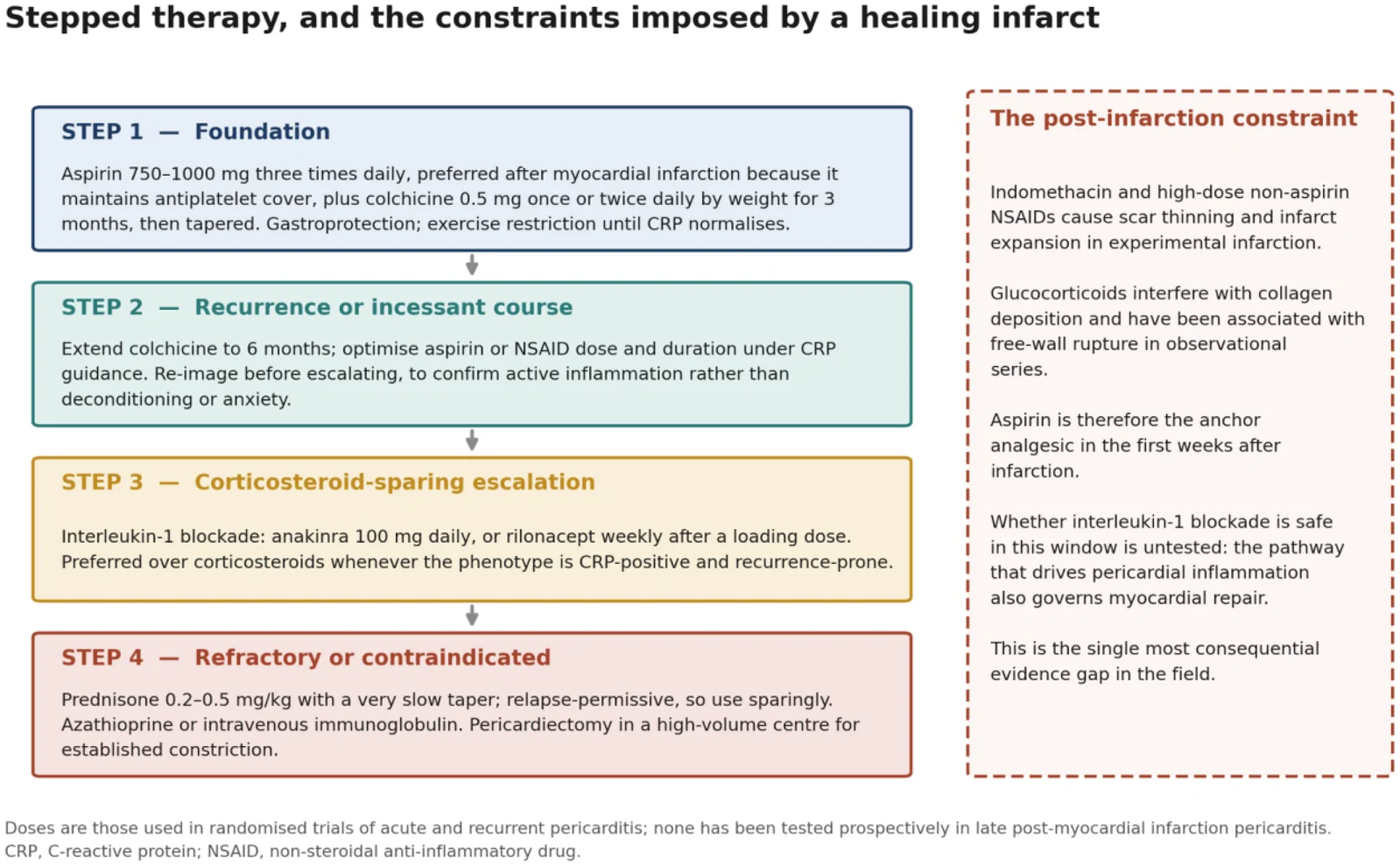
Stepped therapeutic framework, with the constraints imposed by a healing infarct. Doses are those used in randomised trials of acute and recurrent pericarditis; none has been tested prospectively in late post-myocardial infarction pericarditis. CRP, C-reactive protein; IL-1, interleukin-1; NSAID, non-steroidal anti-inflammatory drug.

Colchicine occupies a different evidentiary position from interleukin-1 blockade and should not be described as sharing the same gap. It is the only agent with randomised evidence in a trigger-defined PCIS population, having reduced post-pericardiotomy syndrome in COPPS and COPPS-2 (17, 18), in addition to reducing recurrence in acute and recurrent pericarditis (13–16). Its gap is narrower: no trial has enrolled late post-infarction pericarditis, and the post-infarction trials that established the safety of low-dose colchicine did not record pericarditis as an outcome (47, 48).

What distinguishes post-infarction pericarditis from many other pericardial syndromes is the additional need to consider the biology of infarct healing. In experimental infarction, indomethacin produced marked scar thinning, reducing the ratio of scar to non-infarcted wall thickness from 0.83 to 0.43 (68), and caused infarct expansion with functional deterioration (69). Ibuprofen produced comparable thinning in canine infarction (70). Glucocorticoids impair collagen deposition, and observational data have reported an association between anti-inflammatory treatment and left ventricular free-wall rupture during acute infarction (71). These experimental and observational findings provide a biologically plausible rationale for caution with non-aspirin non-steroidal anti-inflammatory drugs and corticosteroids during the early phase of infarct healing. However, the magnitude and duration of this risk in contemporary reperfused populations are incompletely defined, and the early post-infarction period should not be regarded as a single uniform risk interval.

### 9.1 Interleukin-1 inhibition during infarct healing

Three statements should be kept separate. First, it is biologically plausible that interleukin-1 inhibition could control late post-infarction pericardial inflammation, because the same axis appears central in recurrent pericarditis (Level B) and in experimental pericarditis (Level C) (19). Second, it is also plausible that inhibition during the proliferative phase of repair could alter healing, because interleukin-1 recruits leucocytes, drives a matrix-degrading fibroblast programme and delays myofibroblast conversion until the wound is cleared of debris, and its timely repression is a precondition of effective healing (72); inflammasome activation also promotes adverse remodelling in murine infarction (73). Existing human data do not resolve this: interleukin-1 blockade early after ST-elevation infarction blunted systemic inflammation without an identified safety signal in phase II work (74), and canakinumab reduced recurrent events in patients with prior infarction and residual inflammatory risk (75), but neither enrolled patients with pericarditis and neither was designed to assess scar formation, infarct expansion, remodelling or mechanical complications during active healing. Third, and consequently, interleukin-1 blockade for late post-infarction pericarditis is not an established indication. Its use in this setting is an extrapolation that may be considered in selected refractory, objectively inflammatory cases, and it should be regarded as investigational when initiated during active infarct healing. **Figure 5** illustrates this potential temporal overlap: late post-infarction pericardial inflammation may occur during a period in which myocardial repair remains biologically active. This overlap should be regarded as a hypothesis-generating consideration rather than evidence of harm from interleukin-1 blockade. **Figure 5** illustrates this potential temporal overlap: late post-infarction pericardial inflammation may occur during a period in which myocardial repair remains biologically active. This overlap should be regarded as a hypothesis-generating consideration rather than evidence of harm from interleukin-1 blockade.

**Figure 5:**
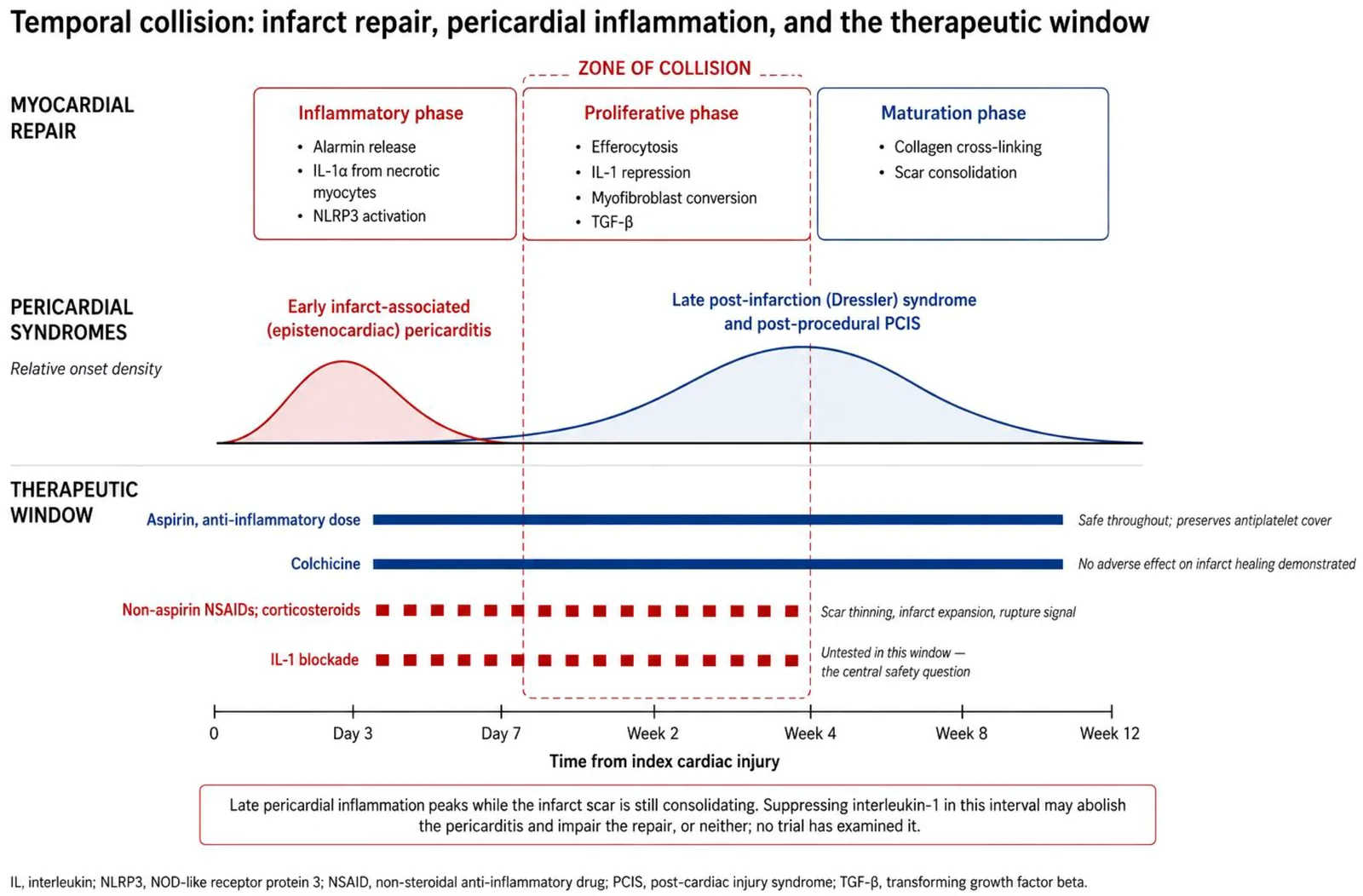
Temporal collision between infarct repair, pericardial inflammation and the therapeutic window. Phase boundaries are approximate, derived from experimental infarct-healing studies scaled to human timelines, and the onset density curves are schematic representations of reported latency distributions rather than empirical distributions. The shaded interval is where the therapeutic decision is actually made and where the evidence is thinnest. IL, interleukin; NLRP3, NACHT, leucine-rich repeat and pyrin domain-containing protein 3; NSAID, non-steroidal anti-inflammatory drug; PCIS, post-cardiac injury syndrome; TGF, transforming growth factor.

### 9.2 Phenotype-guided escalation

Recurrent pericarditis is itself heterogeneous, and this matters for any extrapolation to PCIS. Contemporary practice distinguishes recurrent from incessant disease, and inflammatory phenotypes, with fever, elevated C-reactive protein, effusion or pericardial late gadolinium enhancement, from low-inflammatory presentations in which persistent pain without objective inflammation may not respond to further anti-inflammatory escalation (27, 64, 65). The trials that established interleukin-1 blockade enrolled patients with active recurrence accompanied by systemic inflammation (11, 12), and pericardial enhancement burden predicted recurrence risk in RHAPSODY (76). Interleukin-1 inhibition is therefore supported for selected inflammatory, recurrence-prone phenotypes, not as a validation of interleukin-1 dependency across all pericardial inflammatory syndromes. In PCIS, escalation should be guided by objective evidence of inflammation (biomarkers, imaging, prior therapeutic response and clinical phenotype) rather than by recurrence count alone. The ACC and ESC documents differ in how far they have moved toward such phenotype-driven escalation (26, 27, 77).

### 9.3 Beyond interleukin-1: emerging targets

The therapeutic pipeline for pericarditis now extends beyond established interleukin-1 blockade to direct NLRP3 inhibitors and other targeted agents (78). An oral, peripherally restricted NLRP3 inhibitor is being evaluated in an open-label phase II study in recurrent pericarditis (79). For the purposes of this review, the importance of direct NLRP3 inhibition is mechanistic as much as therapeutic. Anakinra, rilonacept and goflikicept neutralise signalling through the interleukin-1 receptor, which receives input both from inflammasome-processed interleukin-1β and from interleukin-1α released passively from necrotic cells independently of the inflammasome. A response to interleukin-1 blockade therefore cannot distinguish between these sources, whereas a selective NLRP3 inhibitor can. In post-infarction pericarditis, where necrosis-derived interleukin-1α is expected to be abundant, benefit from interleukin-1 blockade without benefit from NLRP3 inhibition would suggest that the alarmin, rather than inflammasome activation, is the dominant driver. No NLRP3 inhibitor has been studied in any PCIS population.

### 9.4 Colchicine in secondary prevention, corticosteroids and supportive care

A second interaction may also be relevant to contemporary epidemiology. Low-dose colchicine reduced ischaemic events after infarction in COLCOT and in chronic coronary disease in LoDoCo2 (47, 48). Where such therapy is prescribed at discharge, some patients may therefore receive a drug that is also established for the prevention of recurrent pericarditis. Neither trial reported pericarditis as an outcome. **Table 7** sets out, trial by trial, what was enrolled and which questions therefore remain unanswered.

**Table 7:** Randomised evidence relevant to Dressler syndrome, and what each trial did not address.

Trial	Population enrolled	Intervention	Principal result	Relevance, and the gap it leaves
COPE, CORP, ICAP, CORP-2	Acute or recurrent pericarditis, mainly idiopathic or viral	Colchicine added to conventional therapy	Consistent reduction in recurrence; ICAP reduced incessant or recurrent disease from 37.5% to 16.7%	Level B evidence; post-infarction cases were not enrolled in meaningful numbers or analysed separately
COPPS, COPPS-2	Patients undergoing cardiac surgery	Perioperative colchicine for prevention	COPPS-2 reduced post-pericardiotomy syndrome from 29.4% to 19.4%, NNT 10; more gastrointestinal adverse events	The only randomised prevention evidence in any PCIS subtype (Level A); no equivalent exists after infarction or ablation
AIRTRIP	Colchicine-resistant, corticosteroid- dependent recurrent pericarditis, n=21	Anakinra versus placebo, randomised withdrawal	Recurrence in 9 of 10 on placebo versus 2 of 11 on anakinra	Proof of efficacy in responders; small, idiopathic, and explicitly not a post-injury population
RHAPSODY	Recurrent pericarditis with systemic inflammation, n=61 randomised	Rilonacept versus placebo, randomised withdrawal	Hazard ratio 0.04; median time to recurrence not reached versus 8.6 weeks on placebo	Most decisive IL-1 evidence (Level B); enriched, idiopathic-predominant, not conducted during infarct healing
CANTOS, COLCOT, LoDoCo2	Post-infarction and chronic coronary disease	Canakinumab; low-dose colchicine	Reduction in recurrent ischaemic events	Both interventions plausibly suppress pericarditis; neither trial reported it as an outcome, so the effect remains unmeasured
None to date	Late post-myocardial infarction pericarditis	—	—	We identified no randomised trial enrolling this diagnosis

IL-1, interleukin-1; NNT, number needed to treat; PCIS, post-cardiac injury syndrome.

Corticosteroids warrant an explicit warning because they remain heavily used — in nearly thirty per cent of patients in one post-ablation cohort (7, 80). They are effective acutely and are associated with a greater risk of recurrence in broader pericarditis populations, particularly when used at high initial doses; when corticosteroids are required, lower doses with gradual tapering are generally preferred (32, 33). In late post-infarction pericarditis, their use should therefore be individualized, taking into account the severity of inflammation, contraindications to alternative therapy, recurrence risk, and the timing of myocardial healing, rather than serving as routine escalation.

Non-pharmacological management is less well defined than pharmacological therapy and should likewise be individualized according to clinical context. Exercise restriction until symptom resolution and normalisation of C-reactive protein is generally recommended during active pericardial inflammation and is particularly reasonable after recent myocardial injury, although direct evidence defining the optimal duration in post-infarction pericarditis is limited. Anticoagulation, frequently mandatory after ablation, device implantation or infarction, deserves an explicit decision rather than reflexive continuation or reflexive interruption, weighed against the risk of haemorrhagic effusion. Pericardiocentesis is indicated for tamponade and where infection is plausible. Pericardiectomy for established constriction belongs in a high-volume centre, and the reported resolution of constrictive physiology with anakinra suggests that anti-inflammatory therapy may be considered in selected patients with potentially reversible, inflammation-associated constriction before surgery, particularly when inflammatory activity persists (81, 82).

## 10 What the existing evidence can and cannot establish

Because so much of the evidence supporting interleukin-1 blockade in recurrent pericarditis rests on two randomised withdrawal trials, it is worth being explicit about what that design does and does not deliver. AIRTRIP and RHAPSODY both enrolled patients who had already demonstrated responsiveness to the study drug during an open-label run-in, and then randomised them to continuation or withdrawal (11, 12). This is a legitimate and efficient design for a relapsing orphan disease, and it produces internally valid estimates of the effect of stopping treatment in known responders. It does not produce an unbiased estimate of the effect of starting treatment in an unselected patient, and the enrichment design may yield larger treatment-effect estimates than would be expected in an unselected first-line population. The hazard ratio of 0.04 reported in RHAPSODY therefore represents a robust treatment effect within a highly selected population of responders, but answers a narrower clinical question than is sometimes implied when the result is extrapolated to other forms of pericarditis.

A second limitation is the dependence of recurrence ascertainment, at least in part, on inflammatory activity. Adjudicated recurrence in these trials typically requires both symptoms and elevated C-reactive protein. Because interleukin-1 blockade suppresses C-reactive protein as part of its biological effect on systemic inflammation, the intervention also modifies one component used to define recurrence. Nevertheless, endpoints incorporating C-reactive protein warrant particular caution when results are extrapolated across disease contexts. This is especially relevant after infarction or surgery, when C-reactive protein rises independently of the pericardium. The third limitation is that patients with PCIS, and particularly with late post-infarction pericarditis, were absent or insufficiently represented for subtype-specific analysis in these trials. When we prescribe an interleukin-1 antagonist for Dressler syndrome, we are performing an extrapolation across aetiology, across the presence of a healing myocardial wound, and across concurrent antithrombotic therapy. Colchicine, with randomised prevention data in post-pericardiotomy syndrome, does not share this gap to the same degree.

## 11 A research programme

The gaps can be stated precisely, which is itself a reason for optimism: many are gaps in evidence generation, harmonisation, and prospective validation rather than evidence of an absent biological framework. We identified no prospectively validated case definition specific to post-cardiac injury syndrome (83); currently used criteria are largely consensus-derived and were developed to capture a heterogeneous group of trigger-defined phenotypes rather than to discriminate among them. Contemporary incidence remains incompletely characterised, in part because pericarditis is not routinely adjudicated as a prespecified adverse event in many infarction and ablation registries. Human mechanistic data specifically obtained in post-cardiac injury pericarditis are also limited. Similarly, validated risk models capable of identifying patients at increased risk of delayed PCIS before discharge are lacking. Finally, interventional evidence specific to the major PCIS phenotypes remains sparse. **Table 8** sets out a programme ordered by feasibility rather than by ambition.

**Table 8:** Prioritised research agenda: gaps, questions, designs and endpoints.

Priority	Gap	Question	Proposed design	Primary endpoint	Feasi- bility
1. Define	Criteria are consensus- based and unvalidated	Does a two-tier clinical and imaging definition perform reliably?	Multicentre prospective validation against CMR-adjudicated reference standard in post-infarction and post-ablation cohorts	Sensitivity, specificity and inter-observer reliability of Tier 1	High
2. Count	Contemporary incidence is inferred, not measured	What is the true incidence by trigger?	Add adjudicated pericarditis to adverse-event schedules of national infarction, ablation and device registries	Incidence per 1,000 procedures by trigger and modality	High
3. Mechanism	No human mechanistic data in PCIS specifically	Does IL-1 activity track disease activity better than antibody titre?	Serial paired plasma and pericardial fluid cytokine profiling indexed to injury magnitude	Correlation of IL-1α and IL-1β with clinical activity score	Moderate
4. Genotype	Inflammasome genotype untested in PCIS	Are gain-of-function variants enriched in recurrent disease?	Case–control targeted sequencing of MEFV, NLRP3, TNFRSF1A, IL1RN	Variant burden, recurrent versus single-episode disease	High
5. Predict	No risk model exists	Who will develop the syndrome?	Derivation and external validation of separate infarction and ablation risk scores	C-statistic and calibration in external validation	Moderate
6. Intervene	No randomised trial has enrolled the diagnosis	Is short-course IL-1 blockade superior to colchicine, and is it safe during scar formation?	Phase II randomised, open-label, blinded-endpoint platform trial with infarction, ablation and surgical strata	Co-primary: recurrence at 6 months; CMR infarct wall thinning at 6 months	Low to moderate
7. Equity	Evidence and access concentrated in high-income reperfusion cohorts	Does classical Dressler syndrome persist where reperfusion is delayed?	Prospective incidence study in South Asian, African and Latin American infarction cohorts	Incidence of Tier 1 PCIS by time to reperfusion	Moderate

CMR, cardiovascular magnetic resonance; IL, interleukin; PCIS, post-cardiac injury syndrome. Feasibility reflects the authors’ assessment of cost, infrastructure and expected recruitment.

The definitional work comes first and is comparatively straightforward. A harmonised, prospectively testable two-tier research definition of the kind proposed in **Table 5** could first undergo derivation and external validation across the major trigger-defined PCIS subtypes. Rather than relying exclusively on new stand-alone cohorts, this work could be embedded within existing infrastructure. Incorporating prospectively adjudicated pericarditis into the adverse-event schedules of national ablation, device, surgical, and infarction registries would permit more robust contemporary incidence estimates while minimising additional data-collection burden.

Mechanistic work is also feasible, but should explicitly distinguish direct human PCIS evidence from extrapolation based on other pericardial syndromes and experimental models. Pericardial fluid is obtained routinely at pericardiocentesis and during cardiac surgery, and is often not subjected to detailed immunological characterisation. Prospective serial profiling of paired plasma and, where clinically obtained, pericardial fluid for interleukin-1α, interleukin-1β, interleukin-18, interleukin-6 and HMGB1, indexed to injury magnitude, could directly test whether the proposed injury–inflammation threshold model is supported in humans. Such studies could be nested within cardiac surgical cohorts, although extrapolation from post-pericardiotomy disease to late post-infarction Dressler syndrome would require caution. Targeted sequencing of MEFV, NLRP3, TNFRSF1A and IL1RN in recurrent versus single-episode post-cardiac injury pericarditis is a plausible hypothesis-generating study, given what has already been found in the idiopathic form (41, 42).

Prediction should then be attempted separately for the infarction and ablation contexts, because the candidate predictors differ: infarct size, transmurality, time to and success of reperfusion and peak C-reactive protein in the first; energy modality, lesion set, procedure duration and age in the second. In parallel, the minimum clinically important difference for pericarditis-specific patient-reported outcomes needs establishing, because clinically meaningful disease burden is driven principally by symptoms, functional limitation, recurrence, and treatment toxicity rather than effusion size alone.

The trial is the hard part, and one candidate research design would be a phase II, randomised, open-label, blinded-endpoint platform design with strata for post-infarction, post-ablation and post-pericardiotomy disease. In the post-infarction stratum, patients meeting the Tier 2 definition could be randomised to short-course interleukin-1 blockade plus aspirin versus colchicine plus aspirin. The primary efficacy endpoint would be recurrence or incessant disease at six months. The co-primary safety endpoint would be change in infarct segment wall thickness and left ventricular end-diastolic volume index on magnetic resonance at six months — thereby prospectively testing, rather than assuming, whether early interleukin-1 inhibition adversely affects infarct healing. The post-infarction stratum should be regarded as an early-phase safety-and-efficacy question rather than as a comparison between two established PCIS strategies. A direct NLRP3 inhibitor arm, once agents mature, would additionally test the inflammasome hypothesis against the interleukin-1α alternative described in Section 9. A platform architecture could permit efficient sharing of statistical infrastructure and adjudication across strata, although control regimens should remain appropriate to each clinical context.

Two cross-cutting commitments belong in the programme rather than in a closing paragraph. The first is access. Anakinra and rilonacept are expensive and unevenly available, and a research agenda that terminates in a biologic risks widening existing disparities in access to effective anti-inflammatory therapy. Establishing whether optimised aspirin and colchicine strategies provide adequate control for a substantial proportion of patients therefore remains an important comparative-effectiveness question, particularly in resource-constrained settings. Oral NLRP3 inhibitors, if effective, could narrow this gap. The second is geography. We identified limited contemporary incidence data from South Asian, sub-Saharan African, and Latin American cohorts. One hypothesis requiring direct testing is that residual late post-myocardial-infarction Dressler syndrome may be more frequent in settings characterised by delayed presentation, incomplete access to reperfusion, failed reperfusion, or larger transmural infarction. Prospective studies with harmonised definitions in geographically diverse infarction cohorts are needed to test it.

## 12 Limitations

This is a narrative state-of-the-art review, and the evidence it synthesises is heterogeneous by necessity; the search described in Section I was designed for transparency, not systematic completeness. The incidence figures derive from cohorts with substantially different case definitions, surveillance strategies, procedural settings, and follow-up periods and are presented without pooling for that reason. The threshold model is a synthesis of existing observations rather than a validated construct, and its worth lies in providing a testable framework and generating falsifiable predictions rather than establishing a specific biological mechanism or mathematical relationship among its components. Evidence incorporated into this framework derives from different levels, including human PCIS studies, studies of non-PCIS pericarditis, experimental models, and hypothesis-generating observations; these are indicated throughout as Levels A, B and C. No component of the innate limb of the model is currently supported at Level A. The role of GATA6-expressing pericardial macrophages, which represents a potentially informative mechanistic hypothesis, remains genuinely contested between two well-conducted murine studies, and direct evidence establishing their role in human PCIS is currently lacking. The proposed trial is a design sketch intended to frame the safety question, not a protocol, and its design assumptions would require refinement before prospective implementation.

## 13 Conclusions

Dressler syndrome has not died; its clinical prominence has diminished in the reperfusion era, while post-cardiac injury syndrome (PCIS) is now encountered across an expanding range of iatrogenic cardiac injuries. The coronary occlusion that once supplied its injury substrate is now interrupted early enough, and often enough, that the classical presentation has become uncommon wherever reperfusion is timely. What has changed is the setting in which the syndrome is recognised, not demonstrably its total burden. The immunological account has moved in parallel: from a predominantly adaptive/autoantibody-based interpretation towards a model in which innate immune activation, including inflammasome and interleukin-1 signalling is proposed to initiate the response while adaptive immunity may amplify its persistence and recurrence. This integrated position rests on evidence from recurrent pericarditis and experimental models, and has not been tested directly in late post-infarction disease.

What has not moved is the evidence base for the eponymous condition itself, which remains, seventy years on, limited and heavily dependent on extrapolation from other forms of pericarditis and from other PCIS subtypes. We propose that whether such inflammation becomes clinically manifest may depend on the interaction between injury burden, the directness of pericardial exposure, local pericardial immune sensing, and host inflammatory susceptibility; this threshold framework is a testable synthesis rather than an established mechanism. Closing that gap requires prospective studies using harmonised case definitions, systematic ascertainment within contemporary registries, and translational sampling of blood and pericardial fluid when clinically available. Equally important is the need for dedicated therapeutic studies in late post-myocardial-infarction pericarditis, particularly to determine the efficacy and safety of interleukin-1 blockade during infarct healing, and to test whether direct NLRP3 inhibition offers a mechanistically discriminating alternative, rather than extrapolating these effects from recurrent pericarditis populations.
